# Prevalence of submicroscopic *Plasmodium falciparum* infections in asymptomatic children in low transmission settings in Bagamoyo, Tanzania

**DOI:** 10.5281/zenodo.10798301

**Published:** 2016-06-17

**Authors:** Deborah Sumari, Joseph Mugasa, Majige Selemani, Seif Shekalaghe, Kefas Mugittu, Paul Gwakisa

**Affiliations:** 1 Ifakara Health Institute, Bagamoyo Branch, Biomedical Thematic group, Bagamoyo, Tanzania; 2 The Nelson Mandela African Institution for Science and Technology, School of Life Sciences and Bioengineering, Arusha, Tanzania; 3 National Institute for Medical Research, Amani Medical Research Centre, P.O. Box 81, Muheza, Tanga, Tanzania; 4 Department of Statistics, University of Dar es Salaam, P.O. Box 35047, Dar es Salaam, Tanzania; 5 Muvek Laboratories, P.O. Box 105270, Dar es Salaam, Tanzania; 6 Genome Science Centre and Department of Veterinary Microbiology and Parasitology, Sokoine University of Agriculture, P.O. Box 3019, Morogoro, Tanzania

## Abstract

**Background:**

Falciparum malaria in endemic areas continues to occur in asymptomatic cases, which contribute to the persistence of transmission as well as the size of the parasite reservoirs. Recent successes in malaria control have resulted in renewed interest in malaria eradication and identification of the human infectious reservoir is essential for this. In this study, we evaluated prevalence of microscopic and submicroscopic gametocytes that were obtained from asymptomatic primary school children from Bagamoyo rural in Tanzania.

**Materials and methods:**

Samples were collected from 501 asymptomatic primary school children (6-14 years of age) from 7 villages in Bagamoyo district. Participants were screened for malaria in the field using RDT, and samples were brought to the laboratory for microscopy and molecular analysis. Parasite density was determined by microscopy, and gametocyte carriage identification was performed by RT-qPCR targeting gametocyte-specific genes.

**Results:**

Asymptomatic infection was found to be 45.1% (95% : CI=40.7-49.6) by RT-qPCR, followed by RDT, 14.2% (95%: CI=11.2-17.5) and microscopy 6.8% (95%: CI=4.7-9.4). Parasite prevalence by microscopy was 12% (23/191) in boys compared to 3.6% (11/310) in girls (p<0.001). Gametocytes were detected in 12.6% (226/501) of the asymptomatic school children by RT-qPCR compared to only 0.8% (4/501) of the children by microscopy (P=0.008).

**Conclusions:**

Asymptomatic infection and submicroscopic gametocyte carriage were high in the study area. The detection of asymptomatic cases with circulating submicroscopic *P. falciparum* gametocytes in school children indicates that these form a substantive gametocyte reservoir that sustains malaria transmission. Asymptomatic carriers and submicroscopic infections should therefore be considered when implementing elimination strategies of the disease.

## 1 Introduction

Submicroscopic *Plasmodium falciparum* gametocytes have been reported in both symptomatic and asymptomatic malaria cases. Asymptomatic individuals constitute a significant reservoir of malaria infection and are important for continued malaria transmission, especially in areas with low endemicity [1]. Asymptomatic infections are not promptly treated due to the fact that they are not diagnosed early [2] and prolonged untreated infections encourage gametocytogenesis [3]. Gametocytes usually develop through five different maturation stages, and those at stage five are microscopically detectable. Their presence in the peripheral blood is a good indicator for characterising the level of transmission in endemic areas [4]. Submicroscopic infection, especially gametocyte carriers, usually constitute an important parasite reservoir in asymptomatic populations [5]. Therefore, at the time when the malaria transmission burden declines across Sub-Saharan Africa, low-level parasitemia present in asymptomatic individuals continues to maintain malaria transmission in affected populations.

Prompt and accurate diagnosis followed by rapid treatment with an effective antimalarial drug is crucial in malaria eradication and elimination. However, the common and standard techniques used for diagnosis of *P. falciparum*, such as microscopy or rapid diagnostic tests (RDTs), are hampered by their low parasite detection limits [6–8]. These tools tend to underestimate *P. falciparum* infection when used alone due to their inability to detect low level parasitemia since malaria transmission is declining in many parts of Sub-Saharan countries [9–12]. Lately, molecular techniques for parasite detection have been introduced to identify parasite densities in epidemiological studies and correctly estimate submicroscopic infection in many laboratories [13–16]. Molecular tools have high sen-sitivity that can assess the extent to which asymptomatic individuals can harbour submicroscopic gametocytes, which are basically underestimated by common diagnostic tools.

The relevance of asymptomatic infections is now being re-evaluated in the light of malaria elimination initiatives, as malaria control focuses not only on clinical malaria but also on identifying and treating asymptomatic malaria infections [12,17]. Malaria elimination is now considered to be an attainable goal for many malaria-endemic regions. Accompanying this shift from malaria control to elimination, interventions are now aimed at targeting all malaria infections that contribute to transmission of the infection, regardless of their symptomatic status [1].

Asymptomatic individuals with submicroscopic gametocytaemia may continue to serve as malaria reservoirs if microscopy and RDT are used alone, due to their low detection limits [18]. However, there are not many data on the prevalence rate of *P. falciparum* infections in asymptomatic cases in Tanzania. Therefore, this study was performed to investigate the prevalence of submicroscopic *P. falciparum* infection in asymptomatic school children in Bagamoyo district, Tanzania, using microscopy, RDT and RT-qPCR.

## 2 Materials and methods

### 2.1 Study design and data collection

A cross-sectional survey was conducted in 501 school children (age 6 – 14 years) between June and December 2014. The study was conducted in Bagamoyo district during two high malaria seasons. The site was selected based on previous reports on low malaria prevalence in this area [19,20]. The Bagamoyo population is approximately 311,740 people, of which 81,000 are village inhabitants [21]. The main rainy season is from March to May, and a short rainy season is from November to December, with an average rainfall of 1200-2100 mm per year. The catchment area is primarily rural with low to moderate malaria transmission [20,22]. Blood samples to determine malaria prevalence were collected from primary school children from Kiwangwa and Yombo wards.

### 2.2 Sample size and sampling procedures

Seven villages (Matimbwa, Yombo, Kongo, Msinune Fukayosi, Mwavi and Kidomole) in Bagamoyo district were selected based on the proximity of the study areas to the laboratory. The selected villages depicted the highest number of children with malaria in the district where 501 school children were enrolled from primary schools from each village. The schools were randomly selected for asymptomatic school children in areas where malaria-positive slide rates were high based on a baseline survey conducted in Bagamoyo villages in 2011/2012 (unpublished data). Sample size was equally distributed in each school but the actual number of participants varied based on the number of parents or guardians that consented, which determined how many children were enrolled from each school.

### 2.3 Screening of participants

Rapid malaria screening was conducted *in situ* at the primary schools. Inclusion criteria were a) presence of informed consent forms, b) asymptomatic malaria status of the child, and c) age 6-14 years. Symptomatic children were referred to a nearby dispensary and were offered appropriate treatment. Those children without symptoms but shown to have body temperature above 37.5°C were further examined for positive blood smears where they were treated with anti-malarial drugs, according to national guidelines.

### 2.4 Malaria screening assays

#### RDT and Microscopy

Three ml of venous blood from each participant were collected into heparin vacutainer tubes (Greiner Bio-One, Kremsmuenster, Austria) for the RDT, microscopy and RT -qPCR assays. The RDT (ICT Malaria Dual Cassette Test; ICT Diagnostics, Cape Town, South Africa) was done on the spot in the field for quick malaria screening as described elsewhere [23]. Thick and thin blood smears from the vacutainer were prepared for microscopy and used to detect and quantify parasites. As a quality control measure two microscopists examined the slides independently. A third microscopist re-examined slides with high discordance. Samples were considered negative if no parasites were detected in 100 high-power fields of Giemsa-stained thick blood smears. Both asexual and sexual stages of the parasites were assessed in thick smears by comparing the ratio of infected to uninfected red blood cells.

Thin smears were examined for parasite speciation and quantification. Gametocytes and asexual stage parasites were counted against 500 and 200 white blood cells (WBCs), respectively, and densities (parasites per micro-liter) were estimated using a factor of 8000 leukocytes/ml. Malaria infection (sexual and asexual stages) prevalence was calculated by a ratio of all positive samples with a total number of participants enrolled.

#### Molecular genotyping

Fifty μl of the sampled blood was spotted onto Whatman 3MM filter paper, air-dried and stored in different plastic-zipped bags together with desiccants, labelled with unique identification number, study number and date. The portion of each dried blood spot (DBS) was cut into 10-12 small pieces and transferred into microcentrifuge tubes containing 250 μl TRizol reagent (Sigma-ALDRICH, St. Louis, USA) and stored at -80°C until RNA extraction.

RNA was extracted from both positive and negative RDT samples collected on 3MM filter papers stored in TRIzol reagent. The filter papers were transferred from TRIzol into 606 μL RLT analysis buffer mixed with β-mercaptoethanol (Qiagen RNeasy plus mini kit) and incubated for 15 min at 30°C on a shaker at 1400 rpm. After incubation, the cocktail was centrifuged for 30 s at 14,000 ***g*** and the aqueous phase was transferred to a gDNA eliminator column, following manufacturer’s instructions with minor modifications. Lastly, RNA was eluted using 50 μL RNase-free sterile water and stored at -80°C for long-term storage.

The *P. falciparum* RNA-based assay for A-type 18S rRNA of the parasite was performed to determine parasite prevalence in a total volume of 12 μL [24]. All positive samples by *P. falciparum* gene expression assay were further analysed for gametocytes prevalence in the same volume. Primers and probes sequences are listed in Table 1. Gametocyte-specific gene *Pfs*25 mRNA transcripts were detected in extracted materials using quantitative nucleic acid sequence reverse transcription polymerase chain reaction (RT-qPCR). *Pfs*25 is expressed only in *P. falciparum* in late stage gametocytes, shows limited polymorphism and is currently used to quantify gametocytes from field isolates [25]. *Pfs*25 transcripts were reverse-transcribed, and the resulting cDNA was amplified by RT-Qpcr, whereas the assay was performed on all extracted RNA samples from asymptomatic individuals after complete gDNA removal. Quantification of *P. falciparum* parasites and gametocytes was calculated in copy numbers obtained of each individual sample per μL of blood using standard curves of the known concentration of the standards. In order to avoid false positive results, a high threshold value of 100 copy numbers was set; all values below this threshold were regarded as false positive.

**Table 1.** Primers and probe sequences used in the study.

Gene	Primers	5’->3’	Probe 5’->3’	Labelled
A-18s	FW	TCCGATAACGAACGAGATCTTAAC	TAGCGGCGAGTACACTATA	FAM-BHQ1
	RV	ATTATAGTTACCTATGTTCAATTTCA		MGB
Pfs 25	FW	GAAATCCCGTTTCATACGCTTG	TGTAAGAATGTAACTTGTGGTAACGGT	HEX-BHQ1
	RV	TGCAGTTTTAACAGGATTGCTTGT		

### 2.5 Data analysis

Data were entered and cleaned in Microsoft Access (Microsoft, Redmond, Washington) and analysed using STATA 11 (StataCorp, College Station, Texas). The analysis used both descriptive and analytical statistics. Malaria prevalence by each village, sex, age and temperature were calculated and presented. Pearson’s Chi-Square test was used to determine the association between a set of explanatory variables and malaria prevalence for categorical variables. Molecular marker specific for parasite copy number/ μL of blood were converted to log_10_.

### 2.6 Ethical considerations

This study received ethical approval from the Institutional Review Board of the Ifakara Health Institute (ethical approval IHI/IRB/No: 34-2013) and the Medical Research Coordinating Committee of the National Institute for Medical Research (No. NIMR/HQ/R.8a/Vol. IX/1705). Regional, district and community authorities in the study area were contacted and granted approval of the study. Prior to participation, sensitisation and mobilisation meetings involving researchers, schoolteachers, children and parents were conducted, where study objectives were explained. Teachers and researchers distributed informed consent forms to the pupils for presenting to parents and/or guardians one week prior to data collection. A written consent of each respondent was obtained based on forms designed for the study. The forms explained risks, benefits and confidentiality associated with the children’s participation in the study. In case a parent or guardian was illiterate, schoolteachers provided assistance to acquaint such par-ents/guardians with the study. Additionally, the confidentiality of all participants was assured by using unique identity study numbers. The parents and/or guardians who consented, signed the informed consent forms and returned these to the study team through head teachers of the schools under study.

## 3 Results

### 3.1 Demographic characteristics of the participants

A total of 501 school children were examined for malaria infection from Yombo and Kiwanga wards (Table 2). Out of 501 children, 191 (38.1%) were males and 310 (61.9%) were females. Study participants aged 6-10 were 190 (37.9%), while those aged 11-14 years were 311 (62.1%). Overall mean age of the children across all schools was 11.1 years.

**Table 2.** Distribution and demographic char acteristics of the study participants.

Ward	Village	*n* (%)	Mean age	Mean Temp (°C)	Mean Weight (kg)
Yombo	Kongo	113 (22.6)	11.5	37.0	33.5
	Matimbwa	51 (10.2)	10.9	36.9	31.4
	Yombo	39 (7.8)	11.4	37.1	29.9
Kiwangwa	Kidomole	136 (27.1)	11.1	36.9	33.2
	Fukayosi	62 (12.4)	10.2	36.6	31.2
	Msinune	57 (11.4)	11.1	36.7	32.9
	Mwavi	43 (8.6)	11.5	36.6	34.1

### 3.2 Prevalence of *P. falciparum* infection in asymptomatic children

Three diagnostic tools, microscopy, RDT and qPCR, were employed to detect the presence of *P. falciparum* infection in the children. Prior to the study, the children were confirmed to be asymptomatic with body temperature below 37.5°C (Table 2).

**Figure 1.**
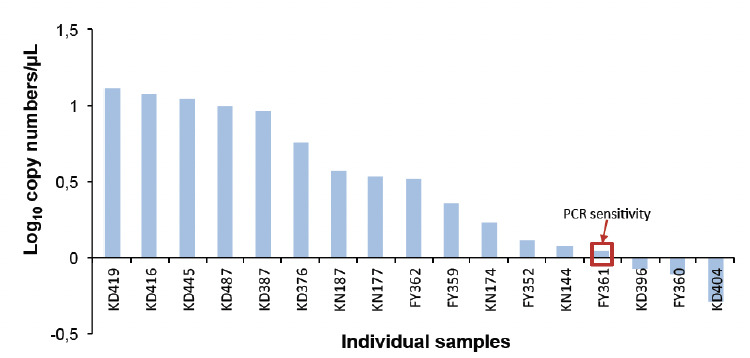
RT-qPCR sensitivity shown by log results of each individual sample.

Prevalence of *P. falciparum* infection by qPCR, RDT and microscopy was 226/501 (45.1%; 95%, CI: 40.7-49.6), 71/501 (14.2%; 95%, CI: 11.2-17.5) and 34/501 (6.8%; 95%, CI: 4.7-9.4), respectively (Table 3). The qPCR assay detected up to 1.11 copy numbers of the *A18S* gene of the *P. falciparum* parasite per μL (Figure 1). The graph was drawn using a log_10_ of copy numbers per μL because the numbers were very diverse. Some samples showed millions of copy numbers while others had a hundred copy numbers. The sensitivity was obtained by repeated experiments to obtain a threshold.

**Table 3.** Prevalence of *P. falciparum* all stages and gametocytes as deter mined by three diagnostic tests (Microscopy, RDT and RT-qPCR); in both cases, n=501.

Village (school)	Microscopy		RDT		RT-qPCR	
	All stages	Gametocytes	All stages	All stages	Gametocytes
Matimbwa	0	0	0	3	1
Yombo	1	0	3	11	1
Kongo	8	0	12	37	12
Msinune	14	1	21	34	20
Fukayosi	1	0	3	31	1
Mwavi	2	1	5	32	10
Kidomole	8	2	27	78	18
Total	34 (6.8%)	4 (0.8%)	71 (14.2%)	226 (45.1%)	63 (12.6%)
CI at 95%	4.7-9.4	0.2-2.0	11.2-17.5	40.7 – 49.6	9.8 – 15.8

Microscopy revealed significantly more positives in boys (12%; 23/191) than in girls (3.6%; 11/310; P<0.001) (Table 4). The proportion of asymptomatic children was also noted between the schools (Table 3). The highest number of asymptomatic children detected with *P. falciparum* infection was indicated by RDT and microscopy tests from Msinune primary school, while the qPCR test showed the highest prevalence in children from Kidomole primary school.

**Table 4.** Malaria prevalence by microscopy, RDT and RT-qPCR against characteristics of participants (n=501).

Characteristics	Microscopy (%)	P-value	RDT (%)	P-value	RT-qPCR (%)	P-value
*Sex*						
Male	23/191 (12%)	<0.001	30/191 (15.7%)	0.439	91/191 (47.6%)	0.371
Female	11/310 (3.6%)		41/310 (13.2%)		135/310 (43.6%)	
*Age group*						
6-10	10/190 (5.3%)	0.289	24/190 (12.6%)	0.440	78/190 (41%)	0.152
11-14	24/311 (7.7%)		47/311 (15.1%)		148/311 (47.6%)	
*Temperature*						
<37.5	30/465(6.5%)	0.284	66/465(14.2%)	0.960	213/465(45.8%)	0.260
*≥37.5*	4/36(11.1%)		5/36(13.9%)		13/36(36.1%)	

When prevalence of *P. falciparum* infection was compared between children aged 6-10 years and those of 11-14 years of age, no significant difference was shown between the age groups, although children in the age group 11-14 years were found to harbour gametocytes significantly more frequent (P=0.008). Detection of asymptomatic *P. falciparum* infections with the three diagnostic methods showed that out of 71 RDT positive samples only 32 were also positive by microscopy (45.1%; 95%, CI: 33.2-57.3). Further, out of 226 qPCR-positive samples, microscopy and RDT showed positive results for 32 (14.2%; 95%, CI: 9.9-19.4), and 54 (23.9%; 95, CI: 18.5-29.9), respectively (Table 5).

**Table 5.** Concor dance in three diagnostic tools for the detection of asymptomatic *P. falciparum* infections; n=501.

A			Microscopy		
			+	-	Total
	qPCR	+	32	194	226
		-	2	273	275
		Total	34	467	501
B			RDT		
			+	-	Total
	qPCR	+	54	172	226
		-	17	258	275
		Total	71	430	501
C			RDT		
			+	-	Total
	Microscopy	+	32	2	34
		-	39	428	467
		Total	71	430	501

### 3.3 Prevalence of *P. falciparum* gametocytes

Gametocyte prevalence was determined by microscopy and RT-qPCR in 501 blood samples. The qPCR method revealed a 63/501 (12.6%; 95%, CI: 9.8-15.8) gametocyte prevalence, while microscopy revealed a much lower prevalence of 4/501(0.8%; 95%, CI: 0.2-2.0; Table 4). Importantly, all gametocyte positive slides were further confirmed by qPCR using the *Pfs*25 gene. Out of 63 qPCR-positive gametocyte samples, 59 (93.7%) were further identified as submicroscopic. In total, 24/63 (38%; 95%, CI: 26.8-50.3) RDT-positive and 37/63 (59%; 95%, CI: 40.7-75.4) microscopy-positive *P. falciparum* infections harboured gametocytes, as determined by qPCR.

## 4 Discussion

We assessed submicroscopic *P. falciparum* infections in asymptomatic school children aged 6–14 years using microscopy, RDT and qPCR. Our results documented a high prevalence of asymptomatic infections among primary school children in the study area. The qPCR method detected a significantly greater number of asymptomatic *P. falciparum* infections compared to the two other methods reported here. Our findings revealed that the qPCR method is more sensitive to detect asymptomatic infections, which concurs with previous studies [15,16,26]. The results show further that the prevalence detected by qPCR was 6.6 and 3.2 times higher than those detected by microscopy and RDT, respectively, indicating that RDT and microscopy grossly underestimate the true prevalence of asymptomatic infections when used alone. Moreover, the observations further emphasise that microscopy is still less sensitive than RDT, as it basically relies on human expertise. However, one shortfall of RDT is the inability of most commercially available kits to differentiate infections from persisting antigens after the infection has been cleared. Despite their limitations, these methods will continue to play a major role in accurate malaria diagnosis. Together with highly sensitive qPCR technique they form key components for successful malaria control.

The prevalence of submicroscopic infections observed in the study area might have a significant impact on malaria transmission, as the asymptomatic state is a major contributor to the infectious reservoir [27,28] and therefore to sustained malaria transmission. The sensitivity of qPCR to detect low-level infections, as has been shown in this study and by others [15,29,30], signifies the need to use qPCR when true estimates of submicroscopic infections are needed for strategic malaria control measures.

Our study revealed a 12.6% prevalence of asymptomatic children with submicroscopic gametocytaemia by qPCR. The role and contribution of the asymptomatic children with submicroscopic gametocytes as reservoirs of malaria infection has not been investigated in the study area, although it is known that submicroscopic gametocytes may contribute to malaria transmission with a similar impact as microscopic gametocytes [25]. The detection of gametocytes carriage from asymptomatic children by microscopy suggests that carriers of submicroscopic gametocytes are common in such settings.

Our findings further demonstrate that, out of all RDT-positive samples, only 45% were positive by microscopy. Despite its advantages, microscopy performance depends entirely on the quality of equipment, expertise of the microscopist, parasite density and time spent to read the slide. Unfortunately, ideal conditions for effective microscopy conduct are rarely met, especially in peripheral health care settings. However, from all qPCR-positive samples, only 14.2% and 23.9% were detected by microscopy and RDT, respectively. Our PCR data helps to confirm the different performance between microscopy and RDT in field studies, which is supported by studies from community surveys [15]. A high sensitivity of qPCR observed in this study to detect submicroscopic infection confirms that the majority of infections in low-transmission settings go unnoticed. However, our results also indicated that the prevalence of asymptomatic infection was significantly associated with sex and age, as female and younger children had a reduced chance of being asymptomatic carriers compared to males and older children, respectively. This finding may be supported by the fact that males have a tendency of harbouring more parasites than females since they are considered to produce more attractive chemicals for mosquitoes than females [31]. Moreover, older children have the striking feature of immune resistance to malaria, which develops after prolonged and sustained exposure to infectious mosquito bites during childhood [32], as was also reported in other studies from Kenya [31], India [33] and Ethiopia [16]. Most children in malaria-endemic areas gain protection against the disease, followed by a decreased rate of symptomatic illness in adolescence [34]. Age is one of the most important factors that correlate positively with protective immunity in malaria-endemic areas. It has been reported that parasitemia in older age groups is lower than the detection limits of conventional malaria diagnostic tools, which therefore fail to detect parasitemia [35].

Identification and management of asymptomatic infections have become crucial challenges for malaria control. Systematic diagnosis of asymptomatic cases as part of surveillance and intervention strategy has been an important phenomenon for reducing parasite reservoirs and helps to decrease malaria. It has been reported that asymptomatic infections can persist in semi-immune individuals for many months if there is no possibility of reinfection [1, 36]. Therefore, a substantial proportion of asymptomatic infection needs to be identified by highly sensitive diagnostic tools for malaria elimination efforts to have better chances of succeeding. This is particularly vital in low-endemic areas where conventional diagnostic tools are most likely to miss these infections.

The strength of this study lies on the fact that it provides a good overview of the malaria burden and infection in an area of low malaria transmission in Tanzania, and it calls for more such studies to be conducted in order to design more effective parasite elimination strategies. However, there were some limitations. The study concentrated on a few villages within an easily accessible geographical area, which restricted our sample size. The study was also bound by limited time and funds; therefore, the submicroscopic gametocyte results from asymptomatic children were not assessed for gametocyte infectivity to mosquitoes. Lastly, most of commercially available RDT kits operate by targeting the presence of circulating antigens; therefore, differentiation of active from resolved infection may be a challenge.

## 5 Conclusions

A large number of submicroscopic *P. falciparum* infection was identified in the study area. Even though malaria transmission in the area is seasonal and unstable, a significant number of asymptomatic infections from school children carrying submicroscopic infection below the threshold of microscopy and RDT were determined. The qPCR method is more sensitive in detecting low levels *P. falciparum* infections and is useful for the correct estimation of malaria prevalence in a given population. The presence of submicroscopic *P. falciparum* gametocytes in this study signals their importance in sustaining malaria transmission in which such infections are relatively common in areas of low to moderate malaria transmission. Although microscopy and RDT detected considerable numbers of asymptomatic infections in apparently healthy children, the use of highly sensitive diagnostics techniques such as qPCR may offer a more accurate assessment and proper estimation of asymptomatic infections. Moreover, the role of submicroscopic parasite carriers in human-mosquito transmission needs to be further determined, especially when malaria control and elimination in Tanzania is to be prioritised.
